# Analysis of IL-1****β**** Release from Cryopreserved Pooled Lymphocytes in Response to Lipopolysaccharide and Lipoteichoic Acid

**DOI:** 10.1155/2013/689642

**Published:** 2013-08-20

**Authors:** Sreelekshmi R. Nair, C. S. Geetha, P. V. Mohanan

**Affiliations:** Toxicology Division, Biomedical Technology Wing, Sree Chitra Tirunal Institute for Medical Sciences and Technology, Thiruvananthapuram, Kerala 695 012, India

## Abstract

Pyrogens are heterogeneous group of fever-inducing substances derived from Gram-positive and Gram-negative bacteria, fungi, and viruses. They incite immune response by producing endogenous pyrogens such as prostaglandins and other proinflammatory cytokines like IL-1**β**, IL-6, and TNF-**α**. The present study was to analyze the influence of cryopreservation in IL-1**β** release, a marker for inflammatory response from human lymphocytes, in response to exogenous pyrogenic stimulants. Lymphocytes isolated from pooled blood of multiple healthy individuals were cryopreserved in DMSO and glycerol for periods of 7, 14, 30, and 60 days and were challenged with LPS and LTA *in vitro*. The inflammatory cytokine, IL-1**β** release, was measured by ELISA method. It was observed that the release of IL-1**β** increases instantaneously after the initiation of incubation and reaches a maximum at 3 to 5 hours and then gradually decreases and gets stabilized for both pyrogens. Moreover it was also observed that the effect of cryoprotectants, DMSO (10%) and glycerol (10%), showed almost similar results for short-term storage, but DMSO-preserved lymphocytes yielded a better viability for long-term storage. Thus, the isolated cryopreserved lymphocytes system can be a promising approach for the total replacement/alteration to animal experimentation for pyrogenicity evaluation.

## 1. Introduction

The inevitable criterion for toxicological safety of any biomaterial, parenteral drugs, cell therapies, recombinant proteins, medical devices, and implants is the conformity with nonpyrogenicity. The manufacturing, processing, and handling of medical products bearing pyrogens on the surface, when brought into human, may lead to inflammatory reactions and reduced biocompatibility [1]. Pyrogenic substances may originate from a variety of biological or synthetic sources. They may also be released from microorganisms such as bacteria, viruses, and fungi during cell lysis or following immunological attack which leads to cell damage or death. One of the most potent pyrogenic materials is lipopolysaccharide (LPS) or otherwise bacterial endotoxin, an outer membrane component of the Gram-negative bacterial cell wall. It has various biological activities and induces fever even in minute amounts [2]. A single *E. coli *contains about two million LPS molecules. Endotoxins are not only released from the cell after lysis but also shed constantly from the live bacteria [3]. Moreover, lipoteichoic acids (LTA) from Gram-positive bacteria are problematic in parenteral pharmaceuticals, since many of the products are prone to contamination by the microbial remnants. Similarly, pyrogenic nature of several biological samples including viruses is not well studied and their mechanism of inducing fever is not well established [4].

The methods used for evaluating pyrogenicity are *in vivo *rabbit pyrogen test and limulus amoebocyte lysate (LAL) assay and monocyte activation. In the conventional rabbit pyrogen test animals were injected with a sample (pharmaceuticals, IV fluids, extract of medical device, etc.) followed by the measurement of rectal temperature. The assay has an inherent problem with respect to the differences in sensitivity towards species [5, 6]. The principle of LAL assay or otherwise bacterial endotoxin test (BET) is based on the extracellular coagulation of the haemolymph of horse shoe crab, *Limulus polyphemus, *when brought into contact with the lipid A portion of endotoxin [7]. The LAL assay is a quantitative assay which detects only Gram-negative bacterial endotoxin and is expensive. It cannot detect any other pyrogens and also there exists a phylogenetic distance between the horseshoe crab and higher vertebrates. Moreover, the dose of bacterial endotoxin eliciting a pyrogenic response varies as much as 10,000 times between species [8]. In order to overcome these limitations, a novel alternative *in vitro* human whole blood assay was developed for detecting pyrogenicity [9].

Exogenous pyrogens like LPS and LTA stimulate monocytes or macrophages and T lymphocytes to produce inflammatory cytokines. These circulating cytokines with endogenous pyrogenic properties reach the organum vasculosum lamina terminalis (OVLT) of central nervous system through fenestrated capillaries [10]. The cytokines may act either directly on neurons or by activating prostaglandins production in OVLT. Prostaglandin E2 is a small lipid molecule which can cross the blood-brain barrier and stimulate the production of neurotransmitters by thermosensitive neurons in the vicinity of circumventricular organs. This will direct the activation of coordinated endocrine, autonomic, and behavioral responses leading to fever [11, 12]. Interleukin 1*β* is one of the main proinflammatory cytokines produced by activated macrophages as a proprotein, which is proteolytically processed into its active form by caspase 1. This cytokine is a main mediator of the inflammatory process, which is immediately released upon exposure to minimal pyrogenic stimulation and can be readily measured by ELISA. Sree Chitra Tirunal Institute for Medical Sciences and Technology developed a rapid, accurate, and cost-effective modified *in vitro* ELISA method for detecting the pyrogenicity of compounds of any nature, which is based on human whole blood [13].

The possibility of obtaining healthy, nonallergic whole blood at the laboratory conditions is little problematic. The use of cryopreserved pooled human blood for detection of cytokine response to lipopolysaccharide has been reported by Megha et al. [14]. IL-1*β* is one of the critical components in the human immune system that fight against infections. Therefore a novel approach was made in the present study using cryopreserved pooled isolated lymphocytes for the evaluation of pyrogenicity using the standardized ELISA method.

## 2. Materials and Methods

### 2.1. Reagents and Materials

Heparin extra pure (Himedia, India), pyrogen-free 0.9% saline (Fresenius Kabi, India) Histopaque-1077 (Sigma, USA), sterile filtered RPMI-1640 (Himedia, India), dimethyl sulfoxide (Me_2_SO, 34869-CHROMASOLV Plus ≥ 99.7%, Sigma Aldrich, USA), Glycerol (SD fine-chem Limited, Mumbai, India), lipoteichoic acid (Sigma, USA), lipopolysaccharide (Sigma, USA), interleukin 1*β* human recombinant expressed in *E. coli* (Sigma, USA), albumin Fraction V from bovine serum (BSA) for biochemistry (Merck, Germany), TMB plus liquid 1 component substrate (Amersco, India), and pyrogen-free 96-well flat-bottom microplates (Nunc Maxisorp, Denmark) were used for ELISA. Purified HRP conjugate anti-IL-1*β* antibody (collected from the rabbits immunized with IL-I*β*) was kindly provided by Toxicology Division, SCTIMST, Thiruvananthapuram.

### 2.2. Equipment

Asys Expert Plus ELISA plate reader (Grenier 96-well ELISA plate) with Digiread software (Austria), Eppendorf Centrifuge 5810R (Germany), Fisher Scientific AB15+ pH meter (UK), ESCO Airstream Vertical Laminar Flow Cabinet (Singapore), Olympus CH-2 microscope (Japan), REMI Centrifuge (India), Heraeus Kelvitron T hot air oven (Germany), and Operon deep freezer −80°C ( Republic of Korea) were used.

### 2.3. Collection of Blood

Healthy, nonallergic volunteers who were not taking any medication for at least one month prior to the blood collection were selected for this study. A total of 80 mL fresh, venous blood was collected from 8 healthy volunteers by venipuncture using sterile syringe and immediately pooled into a sterile, depyrogenated tube containing 40 units of heparin. This blood was used for the isolation of lymphocytes and further for cryopreservation of cells. Informed consent was obtained from the volunteers for the study and was documented.

### 2.4. Isolation of Lymphocytes and Cryopreservation

Lymphocytes were isolated by density gradient centrifugation in Histopaque-1077. The viability of the lymphocytes was assessed with the trypan blue exclusion method. The concentration of cells in the initial mixture was counted and the cells were consequently diluted with RPMI 1640 to obtain a final concentration of 1 × 10^6^ cells/mL. The cells were cryopreserved separately in DMSO (10%) and Glycerol (10%) at −80°C for the periods of 7, 14, 30, and 60 days. This was further used for *in vitro* pyrogen assay.

### 2.5. Induction of IL-1*β* by LPS and LTA in Cryopreserved Isolated Human Lymphocytes with DMSO and Glycerol for Periods of 7, 14, 30, and 60 Days

Cryopreserved lymphocytes at the end of each period were taken and immediately thawed at 37°C. The viability of the cells was calculated before and after cryopreservation in order to obtain the actual count of lymphocytes. The experimental design was shown in Table 1. The lymphocytes were diluted separately in pyrogen-added RPMI-1640 (400 *μ*L) to a final volume of 500 *μ*L in 1.5 mL microfuge tubes. The pyrogens such as LPS from *Escherichia coli *(5 EU) and LTA from *Bacillus subtilis *(1 *μ*g/*μ*L) were used for stimulating pyrogenic response in both the samples. Control experiments were also performed on nonstimulated lymphocytes. The reaction tubes were incubated at 37°C up to 8 hours. At the end of each hour, the samples were centrifuged at 500 g for 2 min at 4°C. The supernatant was transferred to a fresh sterile tube and immediately stored at −20°C until analysis. The same procedure was done with fresh lymphocytes (Table 1) and fresh whole blood (Table 2).

### 2.6. Measurement of IL-1*β* by Sandwich ELISA Method

The anti-human IL-1*β* antibody (collected from the rabbit immunized with IL-I*β*) was diluted with 50 mM carbonate bicarbonate buffer (pH 9.6) coated in a microplate. The plates were kept at 4°C overnight. The antibody-precoated plates were brought to room temperature and blocked with 1% BSA for 1 h at room temperature. The plates were washed using PBS. 50 *μ*L of antigen (supernatant of *in vitro* pyrogen assay) was added per well and incubated at room temperature for 2 h. The wells were washed again and diluted HRP-conjugated anti-IL-1*β* was added and incubated at room temperature for 2 h. TMB substrate was added to the well and incubated for 30 minutes in dark. The reaction was stopped by adding 1 M H_2_SO_4_ and incubated for 10 minutes in dark. The plates were read at 450 nm with the reference at 620 nm using ELISA reader.

## 3. Results

### 3.1. Estimation of IL-1*β* Standards

The IL-1*β* standards were assayed using Sandwich ELISA method. The concentration of IL-1*β* released on stimulation with lymphocytes was calculated from the standard graph by plotting IL-1*β* concentration (ng/*μ*L) on *x*-axis and OD (450 nm) on *y*-axis.

### 3.2. Detection of IL-1*β* Response by Elisa from Fresh Whole Blood, Lymphocytes, and Cryopreserved Lymphocytes for Periods of 7, 14, 30, and 60 Days

**Figure 1:**
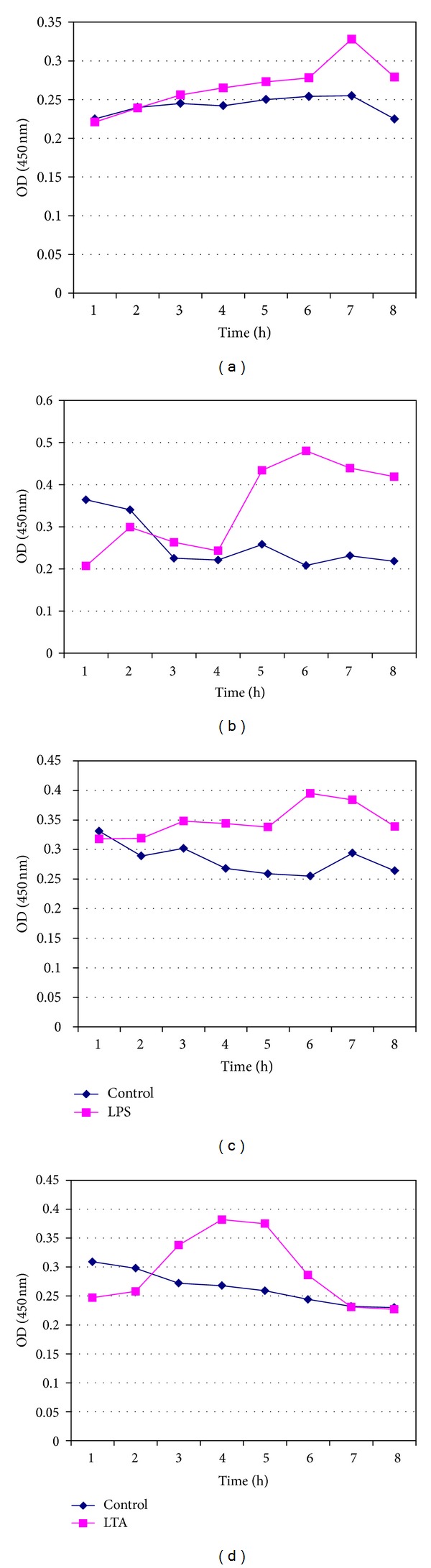
Measurement of IL-1*β* release from (a) fresh whole blood in response to LPS, (b) fresh whole blood in response to LTA, (c) lymphocytes in response to LPS, and (d) fresh lymphocytes in response to LTA.

**Figure 2:**
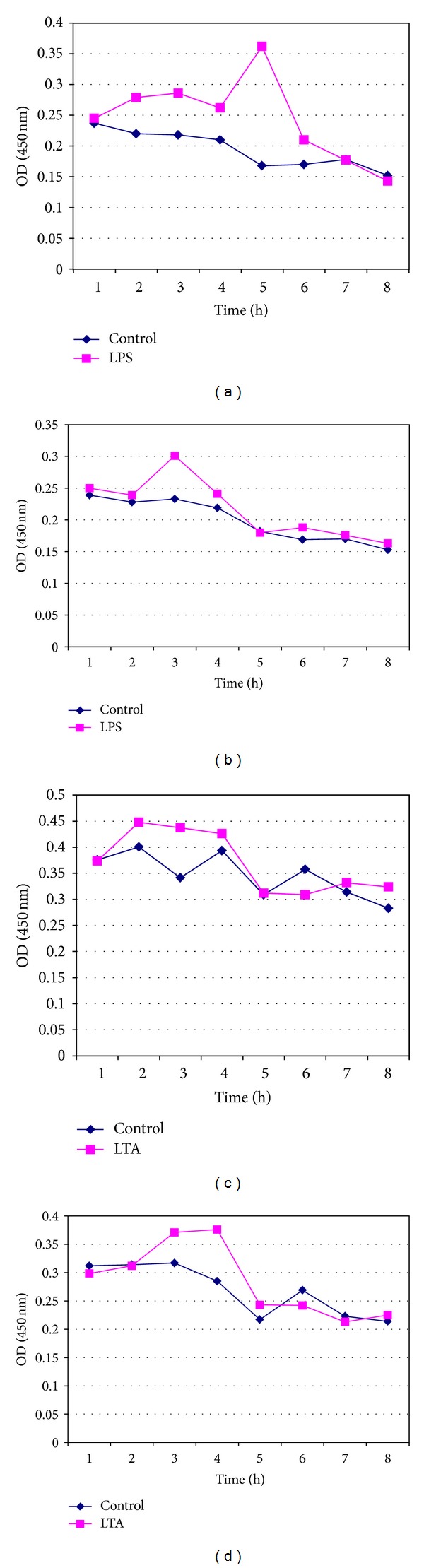
Measurement of IL-1*β* release from pooled isolated 7-day cryopreserved lymphocytes (a) in DMSO in response to LPS, (b) in Glycerol in response to LPS, (c) in DMSO in response to LTA, and (d) in Glycerol in response to LTA.

**Figure 3:**
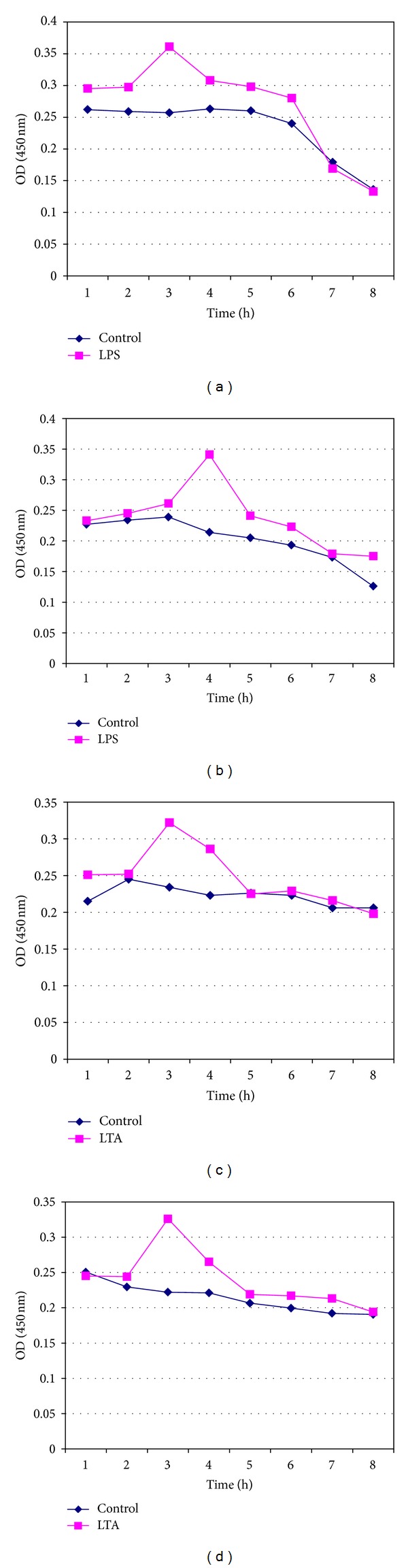
Measurement of IL-1*β* release from pooled isolated 14-day cryopreserved lymphocytes (a) in DMSO in response to LPS, (b) in Glycerol in response to LPS, (c) in DMSO in response to LTA, and (d) in Glycerol in response to LTA.

**Figure 4:**
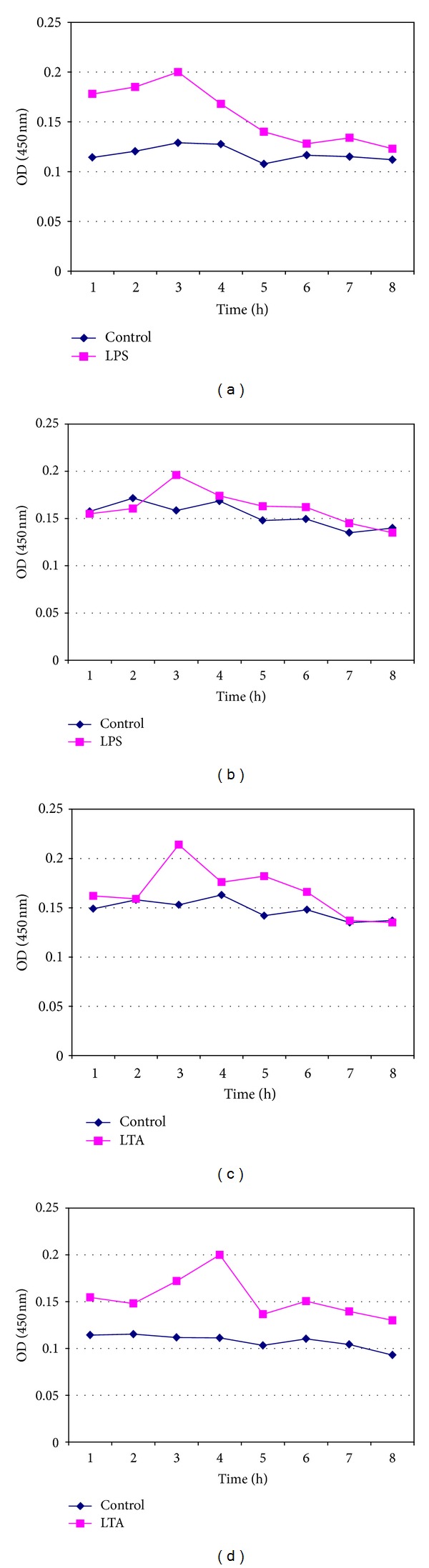
Measurement of IL-1*β* release from pooled isolated 30-day cryopreserved lymphocytes (a) in DMSO in response to LPS, (b) in Glycerol in response to LPS, (c) in DMSO in response to LTA, and (d) in Glycerol in response to LTA.

**Figure 5:**
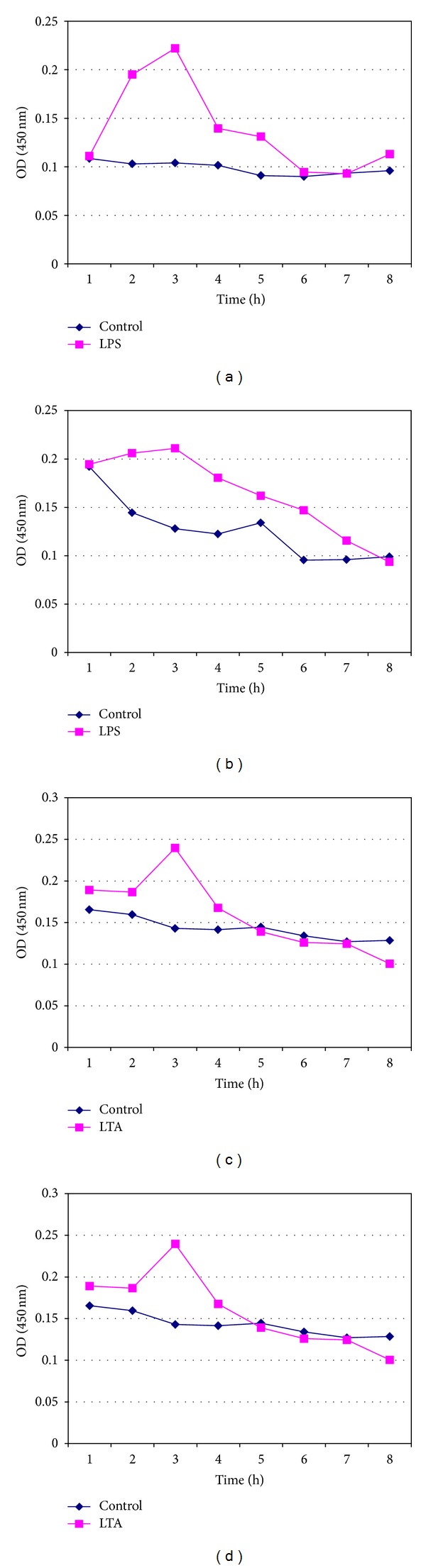
Measurement of IL-1*β* release from pooled isolated 60-day cryopreserved lymphocytes (a) in DMSO in response to LPS, (b) in Glycerol in response to LPS, (c) in DMSO in response to LTA, and (d) in Glycerol in response to LTA.

LPS- and LTA-induced proinflammatory cytokine IL-1*β* was measured from fresh whole blood, fresh lymphocytes (Figures 1(a)–1(d)), and pooled cryopreserved lymphocytes for periods of 7, 14, 30, and 60 days in DMSO and glycerol (Figures 2(a)–2(d), 3(a)–3(d), 4(a)–4(d), and 5(a)–5(d)). This study was conducted for analyzing at what time period the cells are getting activated and stimulated to IL-1*β*.  Table 3 summarizes the time period taken for the interleukin responses.

### 3.3. *In Vitro *Pyrogen Assay with Fresh Whole Blood and Lymphocytes in Response to LPS and LTA

The experimental design of the study was shown in Tables 1 and 2. The time course production of IL-1*β* release in response to 5 EU of LPS and 1 *μ*g/*μ*L of LTA was evaluated for 8 hours. The maximum IL-1*β* production from fresh blood was noted at 7th hour after stimulation with LPS, whereas the maximum release of IL-1*β* was observed between 5 and 7 hours of incubation on challenge with LTA. In fresh lymphocytes, the IL-1*β* release was initiated at 4th hour and the maximum release was observed at 5th and 6th hours of the reaction. In case of lymphocytes stimulated with LTA, the maximum release of IL-1*β* was observed at the 5th hour of reaction (Figures 1(a)–1(d)).

### 3.4. Measurement of IL-1*β* Release from 7-Day Cryopreserved Lymphocytes in DMSO and Glycerol in Response to LPS and LTA

The time course production of IL-1*β* release from lymphocytes (1 × 10^6^ cells/mL) cryopreserved in DMSO and Glycerol for a period of 7 days, when stimulated with 5 EU of LPS and 1 *μ*g/*μ*L of LTA, was evaluated for 8 hours. The maximum release of IL-1*β* was observed at 5th hour for the cryopreserved lymphocytes in DMSO, whereas in case of cells preserved in glycerol it was found to be maximum at 3rd hour, when stimulated with LPS. For cells stimulated with LTA, the maximum IL-1*β* level was noted at 3-4 hours of incubation, while the maximum IL-1*β* level for the cells cryopreserved in glycerol was found at 4th hour of incubation (Figures 2(a)–2(d)).

### 3.5. Measurement of IL-1*β* Release from 14-Day Cryopreserved Lymphocytes in DMSO and Glycerol in Response to LPS and LTA

The maximum IL-1*β* production in the reaction when induced with LPS was observed at 3rd and 4th hours of incubation for the lymphocytes cryopreserved in DMSO and Glycerol, respectively, and for LTA-stimulated cryopreserved lymphocytes the peak was observed at 3rd hour of the reaction (Figures 3(a)–3(d)).

### 3.6. Measurement of IL-1*β* Release from 30-Day Cryopreserved Lymphocytes in DMSO and Glycerol in Response to LPS and LTA

In pooled lymphocytes cryopreserved in DMSO and glycerol, the IL-1*β* release elicited by LPS and LTA was clearly observed. There is an increase in IL-1*β* release at the 1st and 2nd hours of incubation and the maximum release was observed at the 3rd to 4th hours (Figures 4(a)–4(d)). As observed in all other cases, IL-1*β* release increased during the induction of endotoxin in the first few hours and then slowly decreased and stabilized at the end of the 8th hour.

### 3.7. Measurement of IL-1*β* Release from 60-Day Cryopreserved Lymphocytes in DMSO and Glycerol in Response to LPS and LTA

**Figure 6:**
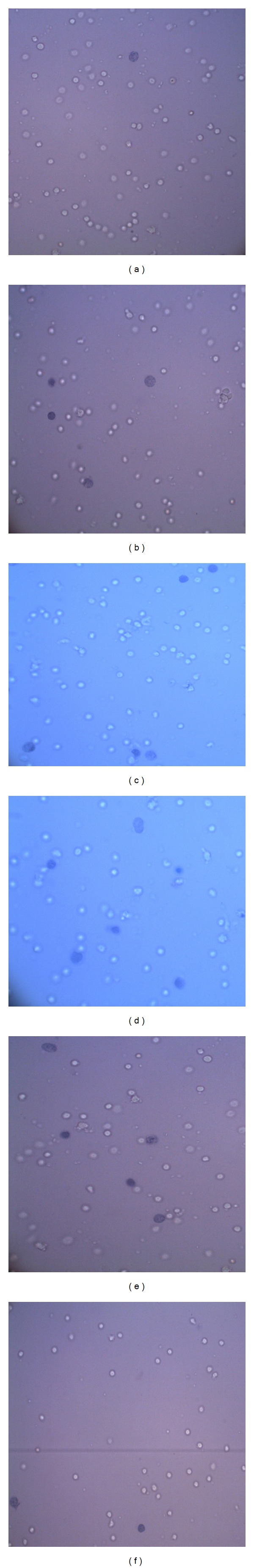
Cell viability study of (a) DMSO-added fresh lymphocytes, (b) Glycerol-added fresh lymphocytes, (c) 7-day cryopreserved lymphocytes in DMSO, (d) 7-day cryopreserved lymphocytes in Glycerol, (e) 60-day cryopreserved lymphocytes in DMSO, and (f) 60-day cryopreserved lymphocytes in Glycerol.

The maximum level of IL-1*β* release was observed within the 3rd hour and 4th hour, when 60-day cryopreserved (both in glycerol and DMSO) lymphocytes were stimulated by LPS and LTA (Figures 5(a)–5(d)). When compared to the first 7 days, the viability of the cells decreased gradually. Thus the responses of cells were reduced in comparison to the 1st and 2nd weeks (Figures 6(a)–6(f)).

## 4. Discussion

Human growth hormone is biosynthetically produced in recombinant strains of *Escherichia coli* as methionyl human growth hormone (met-hGH). When purified from the bacterial culture, met-hGH is biologically active in established assays for growth hormone. Studies demonstrated negative result from the LAL and rabbit pyrogen test in the met-hGH which induced acute-phase reactions. In addition, this study demonstrated that the release of leukocytic pyrogen (LP) from human cells is a reliable indicator of the presence of materials that are pyrogenic for humans [15].

Recombinant growth hormone administration reduced urea generation and improved the efficiency of dietary protein utilization in stable adult hemodialysis patients. Growth hormone may be a useful adjunctive therapy to diminish body protein catabolism in this patient population. Recombinant human methionyl growth hormone (Protropin) (Genetech, Inc., San Francisco, CA, USA) caused significant and sustained nitrogen retention over a wide range of nutritional support. GH enhanced the efficacy of parenteral nutrition in stable individuals requiring repletion of body protein [16, 17].

Macrophages and mononuclear phagocytes are known to produce and release a variety of cytokines, including interleukins (IL) and tumor necrosis factor (TNF), in response to endotoxin stimulation both *in vivo *and *in vitro*. These cytokines mediate the harmful effects of the endotoxins *in vivo* leading to endotoxemia which results in septic shock and multiple system organ failure [18].

Cytokines are one of the major signaling molecules mediating inflammatory reactions. The interaction with cytokines depends on a variety of factors like species, mode of infection, and strength of fever inducing stimulus. Cytokines show pleiotropism, redundancy, and feedback mechanisms [19]. The key role of proinflammatory cytokines like IL-1*β* in inducing pyrogenic response necessitates the development of assays for cytokine quantification which becomes a rapidly expanding part of the laboratory analysis. IL-1*β* is a potent proinflammatory cytokine secreted by blood monocytes and tissue macrophages, when they come in contact with exogenous pyrogens [20]. It has an advantage as a read-out parameter for Gram-negative and Gram-positive pyrogens because of the shorter incubation time required for its release [21].

The selection of a suitable cytokine assay depends to a large extent on the research objective to be achieved. In the present study an effort was made to isolate lymphocytes from pooled human blood and they were cryopreserved in 10% DMSO and 10% Glycerol for periods of 7, 14, 30, and 60 days to evaluate the release of IL-1*β*, as a marker for pyrogenicity induced by LPS, (5 EU) and LTA (1 *μ*g/*μ*L) using enzyme-linked immunosorbent assay (ELISA).

In order to study the influence of cryopreservation on cell viability and IL-1*β* release, control cells (without LPS and LTA treatments) and test samples (with LPS and LTA treatments) were used. The cryopreserved lymphocytes stored at −80°C for periods of 7, 14, 30, and 60 days were monitored for IL-1*β* release and it was observed that the maximum release of IL-1*β* was at 3 to 5 hours in both LPS and LTA treatments.

The amount of endotoxin range required to generate a pyrogenic reaction is 0.5 EU–5 EU and the threshold value for fever induction is 0.5 EU [22]. The present study suggests that the time course of IL-1*β* production after stimulation with LPS and LTA corresponds to the time course of fever induction.

The time course production of IL-1*β* release in response to LPS (5 EU) was evaluated for 8 hours. The maximum release of IL-1*β* was observed at 5th hour for the one-week (7-day) cryopreserved lymphocytes in 10% DMSO, whereas the release of IL-1*β* level was found maximum at the 3rd hour, when treated with lymphocytes cryopreserved in glycerol. It was also observed that the maximum IL-1*β* level in the *in vitro *reaction using LTA (1 *μ*g/*μ*L) was at 3-4 hours of incubation using 7-day cryopreserved lymphocytes in 10% DMSO, while the maximum IL-1*β* level for the cells cryopreserved in 10% glycerol was found at 4th hour of reaction.

The stimulation with Gram-negative endotoxin (LPS) gave a maximum response in IL-1*β* production at the 3rd hour of the reaction, whereas the maximum release of IL-1*β* level of lymphocytes cryopreserved with glycerol was found at the 4th hour, when the lymphocytes were cryopreserved in DMSO for 2 weeks (14 days). Correspondingly the maximum IL-1*β* production level was observed at 3rd hour for both the reactions when LTA was added.

The IL-1*β* release elicited by 5 EU of LPS was observed maximum at the 3rd hour of the reaction when the lymphocytes (DMSO and Glycerol) were cryopreserved for a period of one month (30 days). There was a significant increase in the IL-1*β* release in the first 2 hours reaching the maximum at the 3rd hour. On the other hand the IL-1*β* response elicited by 1 *µ*g/*µ*L of LTA was shown to have a maximum peak at 3-4th hours, respectively. 

It was noted that the maximum level of IL-1*β* release was within 3 to 4 hour, when exposed to cryopreserved (60 days, both glycerol and DMSO) lymphocytes on LTA and LPS. As observed in all other cases, IL-1*β* release increased during the induction of endotoxin in the first few hours and then slowly decreased or stabilized at the end of the 8th hour and these findings are similar to the findings of Banerjee and Mohanan, in 2011 [4].

The viability of the cells decreased gradually during prolonged period (1 and 2 months) of cryopreservation, but this reduction was not found to affect the IL-1*β* release on challenge with pyrogens. It was confirmed from our earlier studies [23] that approximately 5 × 10^5^ cells were required for the reaction. Consequently, the cellular responses (viability) against pyrogens were relatively low when compared to the 1st and 2nd weeks. These inconsistency results may be due to the inhibitory factors affecting the IL-1*β* release as supported by the finding of Dinarello in 2004, which stated that the steady-state levels of IL-1*β* increase with transient transcription and decreases due to the synthesis of a transcriptional repressor.

The effect of cryoprotectant on maintaining cell viability was also studied. After 30 days of cryopreservation approximately 70% of cells were found to be alive with DMSO as a cryoprotectant, whereas in case of glycerol only 50% of cells were viable. Even though both DMSO and glycerol gave almost similar results for short-term storage, the DMSO-preserved lymphocytes yielded a better viability for long-term storage.

The in-house developed ELISA [22] for the detection of IL-1*β* requires minimal incubation time and is based on Sandwich method of antigen detection [24, 25]. The advantage of cryopreserved lymphocytes over fresh human whole blood and fresh lymphocytes is the off-shelf availability of screened, pathogen-free, and allergen-free system for IL-1*β* detection. The results of the present study indicated that there was a delay in the IL-1*β* release in whole blood compared to fresh lymphocytes and cryopreserved lymphocytes. This may be due to the presence of other components in blood that interfere with IL-1*β* production. It was also demonstrated that the morphology and physiological functions of the cells are highly preserved after cryopreservation. The cryopreserved lymphocyte test system can be used to detect Gram-negative and Gram-positive pyrogens, since it measures the IL-1*β* release even with small stimulations. This in turn depends on the percentage of viable cells. These findings were supported by our previous studies [21, 23].

When using the lymphocyte test system, it was observed that the difference in blood group does not interfere with IL-1*β* release having an added advantage of using pooled blood. These findings were similar to the report of Mazzotti et al. in 2007 [26] that the large interindividual variation in case of the cryopreserved blood is reduced by pooling blood from at least five donors before freezing. Thus, the isolated cryopreserved lymphocytes system can be a promising approach for the total replacement/substitution of animal experimentation for pyrogenicity evaluation.

## Figures and Tables

**Table 1:** Experimental design for *in vitro* pyrogen testing.

Observation periods (days)	Fresh blood (single donor) (hrs)		Fresh lymphocytes (h) (single donor)		Pooled lymphocytes cryopreserved in DMSO (h)		Pooled lymphocytes cryopreserved in Glycerol (h)	
	Pyrogens							
	LPS (5 EU)	LTA (1 *μ*g/*μ*L)	LPS (5 EU)	LTA (1 *μ*g/*μ*L)	LPS (5 EU)	LTA (1 *μ*g/*μ*L)	LPS (5 EU)	LTA (1 *μ*g/*μ*L)
0	7	5 and 7	5 and 6	5	0	0	0	0
7					5	3 and 4	3	4
14					3	3	4	3
30					3	3	3	4
60					3	4	3	3

**Table 2:** Cytokine (IL-1*β*) induction by LPS and LTA in fresh blood.

Components	Control	LPS (EU)	LTA (*µ*L)
RPMI (*μ*L)	400	395	390
Cells (*μ*L)	100	100	100
Pyrogen	—	5	10

Total volume (*μ*L)	500	500	500

**Table 3:** IL-1*β* release from fresh blood, fresh lymphocytes, pooled isolated cryopreserved lymphocytes in DMSO and Glycerol with response to LPS and LTA.

Components	Control	LPS (EU)	LTA (*μ*L)
RPMI (*μ*L)	600	600	600
Saline (*μ*L)	100	95	90
Blood (*μ*L)	300	300	300
Pyrogen	—	5	10

Total volume (*μ*L)	1000	1000	1000
